# PTH infusion ameliorates seizures in autosomal dominant hypocalcemia type 1

**DOI:** 10.1056/NEJMc2034981

**Published:** 2021-07-08

**Authors:** Ana Sastre, Kevin Valentino, Fadil M. Hannan, Kate E. Lines, Anna K. Gluck, Mark Stevenson, Michael Ryalls, Debbie Pullen, Jackie Buck, Sailesh Sankar, Jeremy Allgrove, Rajesh V. Thakker, Evelien F. Gevers

**Affiliations:** Department of Paediatric Endocrinology, Barts Health NHS Trust – Royal London Children’s Hospital, London, United Kingdom; Centre for Endocrinology, William Harvey Research Institute, Barts and the London School of Medicine and Dentistry, Queen Mary University of London, United Kingdom; Academic Endocrine Unit, Radcliffe Department of Medicine, University of Oxford, Oxford, United Kingdom; Nuffield Department of Women’s & Reproductive Health, University of Oxford, Oxford, United Kingdom; Academic Endocrine Unit, Radcliffe Department of Medicine, University of Oxford, Oxford, United Kingdom; Academic Endocrine Unit, Radcliffe Department of Medicine, University of Oxford, Oxford, United Kingdom; Academic Endocrine Unit, Radcliffe Department of Medicine, University of Oxford, Oxford, United Kingdom; Royal Surrey County Hospital, Guilford, United Kingdom; Surrey and Sussex Healthcare NHS Trust-East Surrey Hospital, Surrey, United Kingdom; East Suffolk and North Essex NHS Foundation Trust - Ipswich Hospital, Ipswich, United Kingdom; Department of Endocrinology and Diabetes WISDEM Centre, University Hospitals Coventry and Warwickshire NHS Trust, Coventry, United Kingdom; Department of Paediatric Endocrinology, Barts Health NHS Trust – Royal London Children’s Hospital, London, United Kingdom; Department of Paediatric Endocrinology, Great Ormond Street Hospital, London, United Kingdom; Academic Endocrine Unit, Radcliffe Department of Medicine, University of Oxford, Oxford, United Kingdom; Centre for Endocrinology, William Harvey Research Institute, Barts and the London School of Medicine and Dentistry, Queen Mary University of London, United Kingdom; Department of Paediatric Endocrinology, Barts Health NHS Trust – Royal London Children’s Hospital, London, United Kingdom

TO THE EDITOR: Autosomal dominant hypocalcemia type 1 (ADH1) is caused by gain-of- function calcium-sensing receptor (CaSR) mutations^1,2^ that result in hypocalcemia and seizures, hypomagnesemia, hyperphosphatemia, reduced parathyroid hormone (PTH), and hypercalciuria. Calcium and vitamin D analogs for treating ADH1 predispose to nephrocalcinosis and renal impairment^1,3^. CaSR antagonists, known as calcilytics, represent a possible treatment^2^, but are clinically unavailable. However, recombinant PTH(1-34) may increase serum calcium without causing hypercalciuria in patients with forms of hypoparathyroidism^4,5^. We assessed the effectiveness of continuous subcutaneous PTH(1-34) infusion (CSPI) in a retrospective cohort of six ADH1 patients (aged 5 weeks-22 years), who were selected as they experienced hypocalcemic seizures despite using calcium and vitamin D analogs, and/or bolus PTH injections (Fig. 1A and Methods section in the Supplementary Appendix). Calcium and vitamin D analogs also failed to cease anticonvulsant therapy in two of three patients on phenobarbitone, and were associated with nephrocalcinosis and renal impairment in two patients, respectively. All patients had gain-of-function CaSR mutations, with three being constitutively active mutations that had arisen *de novo* and showed diminished signalling responses to the calcilytic, NPS-2143 (Fig. 1A, Fig. S1, Fig. S2 and Methods section in the Supplementary Appendix). CSPI treatment over 0.8-5.5 years increased mean serum adjusted-calcium by 0.30mmol/L (95% confidence interval (CI), 0.12 to 0.48) and reduced mean serum phosphate by 0.92mmol/L (95%CI, 0.69 to 1.14) in all six patients, when compared to calcium and vitamin D analog treatment (Fig. 1B). This was associated with decreased mean calcium-phosphate product by 1.15mmol^2^/L^2^ (95%CI, 0.63 to 1.66) in 5 patients, and increased mean serum magnesium by 0.09mmol/L (95%CI, 0.03 to 0.14) in 4 patients (Fig. 1B). CSPI reduced seizures in all patients from 2.0 (95%CI, -1.6 to 5.6) to 0.01 (95%CI, -0.01 to 0.02) seizures per month, with no patients requiring further anticonvulsants, and resulted in fewer emergency admissions (Fig. 1C). Serious adverse effects were not observed during CSPI. Tachyphylaxis was suspected in one patient with a slipped upper femoral epiphysis, but bone mineral apparent density in three children remained within the reference interval, and CSPI did not worsen nephrocalcinosis or increase calcium excretion, which was reduced in three patients (Table S1, Fig. S3 and Fig. S4 in the Supplementary Appendix). All CSPI-treated infants attained developmental milestones. Thus, CSPI represents a long-term therapy for increasing serum calcium, ameliorating seizures and reducing hospital admissions in young ADH1 patients. A prospective study is required to confirm these findings.

## Supplementary Material

Supplementary Appendix

## Figures and Tables

**Figure 1:**
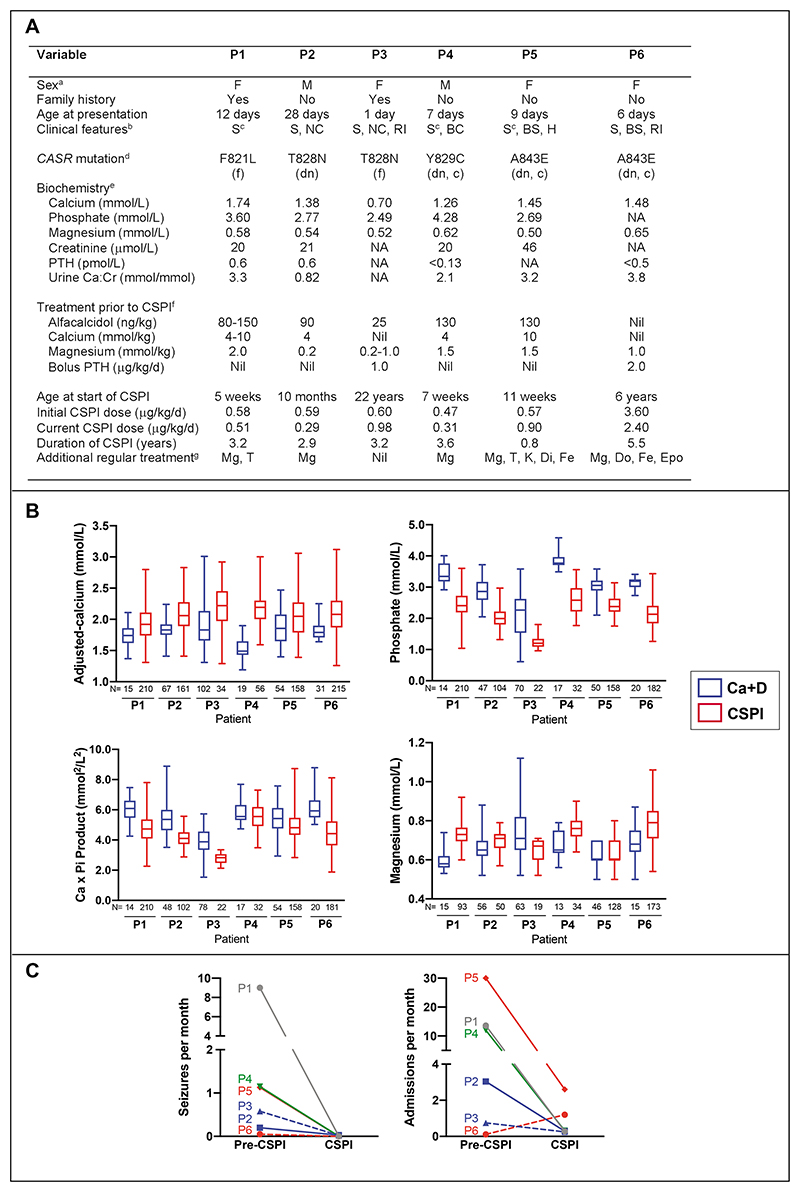
Clinical findings and responses to PTH infusion in ADH1 patients. Panel A shows clinical characteristics and treatments for all six unrelated ADH1 patients (P1-P6). ^a^M, male; F, female. ^b^S, seizures; NC, nephrocalcinosis; RI, renal impairment; BC, bilateral cataracts; BS, Bartter syndrome; H, hyperinsulinism. ^c^Received phenobarbitone anticonvulsant therapy. ^d^f, familial; dn, *de novo;* c, constitutively active. ^e^Normal ranges: adjusted-calcium, 2.20-260 mmol/L; phosphate, 1.30-2.60 mmol/L (<1 month), 1.30-2.40 mmol/L (1 month-1 year), 0.90-1.80 mmol/L (1-16 years), 0.80-1.50 mmol/L (>16 years); magnesium, 0.70-1.0 mmol/L; creatinine, 27-77 μmol/L (<1 month), 14-34 μmol/L (<1 year), 15-31 μmol/L (1-3 years), 23-37 μmol/L (3-5 years), 25-42 μmol/L (5-7 years), 30-47 μmol/L (7-9 years), 29-56 μmol/L (9-11 years), 39-60 μmol/L (13-15 years), 40-68 μmol/L (>15 years); parathyroid hormone (PTH) 1.6-6.9 pmol/L; urine calcium: creatinine (Ca:Cr) ratio, <1.50 mmol/mmol (0-1 years), <1.25 mmol/mmol (1-2 years), <1.0 mmol/mmol (2-5 years), <0.70 mmol/mmol (5-10 years), <0.60 mmol/mmol (10-18 years). ^f^CSPI, continuous subcutaneous PTH(1-34) infusion; NA, not available; ^g^Mg, magnesium; K, potassium; T, thiazide, Di, diazoxide; Do, doxazosin; Fe, ferrous fumarate; Epo, erythropoietin. All children were taking cholecalciferol or ergocalciferol. Panel B shows effects of CSPI compared to prior therapy with oral calcium supplements and vitamin D analogs (Ca+D) on serum concentrations of adjusted-calcium; phosphate; calcium-phosphate product (Ca x Pi); and magnesium. Number of serum biochemical values per treatment group in each patient (P1-P6) are shown below respective box and whisker plot. Panel C shows effects of CSPI compared to prior therapy with Ca+D (Pre-CSPI) on numbers of seizures per month and hospital admissions per month, in six ADH1 patients (P1-P6).
