# Supplementary material for: PTH infusion ameliorates seizures in autosomal dominant hypocalcemia type 1
Source: N Engl J Med. Author manuscript; Available in PMC 2023 Aug 1. (PMC7614858; doi:10.1056/NEJMc2034981)
Supplement: Supplementary Appendix [file EMS181864-supplement-Supplementary_Appendix.pdf]

Supplementary Appendix

This appendix has been provided by the authors to give readers additional information about their work

Supplement to Sastre A., Valentino K., Hannan F.M., Lines K.E., Gluck A.K., Stevenson M., Ryalls M., Pullen, Buck J., Sankar S., Allgrove J., Thakker R.V., Gevers E.F. PTH infusion ameliorates seizures in autosomal dominant hypocalcemia type 1.

Table of Contents

METHODS...3

Patients……………………………………………………………………………..3
Administration of rhPTH(1-34) by continuous subcutaneous infusion…………………9
Monitoring…………………………………………………………………………10
DNA sequence analysis……………………………………………………………10
Cell culture and transfection……………………..10
Luciferase reporter assays... 12
Statistical analysis…………………………………………………………………12

TABLES...13

Table S1...14

FIGURES...15

Figure S1...15
Figure S2...16
Figure S3...18
Figure S4...20

REFERENCES………………………………………………………..............21

METHODS

Patients

Six patients with hypocalcemia, low PTH concentrations and recurrent seizures, who were inadequately managed on oral calcium and vitamin D analogs, magnesium supplementation, and/or bolus recombinant PTH injections, were investigated in this retrospective observational study. All of the patients in this study have a severe form of ADH1 characterised by neonatal onset of hypocalcemic seizures, and which is associated with germline *CASR* mutations that cause constitutive or non-constitutive increases in CaSR activity. Some of these CaSR mutations, in addition to causing marked disturbances in calcium homeostasis, were associated with non-calcitropic phenotypes. Thus, patient 5, who harbours a constitutively activating CaSR mutation, p.Ala843Glu, experienced hypoglycemia due to endogenous hyperinsulinism, which may be a consequence of activation of the CaSR within pancreatic beta cells, as the pancreatic islet CaSR has been previously shown to regulate insulin secretion[1,2]. Furthermore, patient 4 developed bilateral cataracts in infancy, and such findings have been reported in an ADH1 mouse model[3], although the cause of cataracts in ADH1 remains to be elucidated. Their clinical and biochemical features are described in greater detail below.

Patient 1

Presentation: Patient 1 was born at 37 weeks gestation with a birth weight of 1.95 kg. Parents are of Indian origin. Pregnancy was complicated by prelabor rupture of membranes (PROM), for which the mother received antibiotics. The patient had isolated hypoglycemia in the neonatal period (plasma glucose: 2.1 mmol/L, normal range 2.6-7.8 mmol/L), and presented with hypocalcemic seizures at 12 days of age. Her father had hypoparathyroidism and required dialysis at 17 yrs of age and renal transplantation at 25 yrs of age for nephrocalcinosis and

chronic kidney disease (CKD). He had undiagnosed ADH1. Patient 1 had hypomagnesemia, hyperphosphatemia, low circulating PTH concentrations, and hypercalciuria at diagnosis (Fig. 1A). She required multiple i.v. boluses of calcium and magnesium which provided temporary amelioration of the seizures, and she was treated with phenobarbitone for 2 weeks, which was discontinued after her serum calcium had normalised on CSPI, following which she remains free of seizures.

Before CSPI: She was treated with high dose oral alfacalcidol, cholecalciferol, calcium and magnesium supplementation, and i.v. boluses and infusion of calcium (Fig. 1A). However, her seizures persisted.

After CSPI: She started CSPI at 5 weeks of age, which normalised the serum calcium and abolished the seizures. She required short admissions for asymptomatic hypocalcemia due to cannula failure or intercurrent illnesses. Her serum creatinine has remained stable on CSPI and there is no evidence of nephrocalcinosis. She was walking and talking at an appropriate age, and is growing normally. Bone mineral apparent density (BMAD) at the age of 3 yrs is within the age-adjusted pediatric reference interval (Fig. S4).

Patient 2

Presentation: Patient 2 was born at 35 weeks gestation with a birth weight of 3.01 kg, and presented with bronchiolitis and seizures at 28 days of age. He had hypocalcemia, hypomagnesemia, hyperphosphatemia, and low circulating PTH concentrations (Fig. 1A). His parents are of White European origin. There was no family history of hypocalcemia.

Before CSPI: He was treated with oral alfacalcidol, and calcium and magnesium supplementation (Fig. 1A), and was admitted to hospital 1-2 times per week with symptoms of hypocalcemia (tetany). He also had mild polyuria and polydipsia, consistent with hypercalciuria, and by the age of 10 months had developed bilateral severe nephrocalcinosis.

After CSPI: He started CSPI treatment at 10 months of age. He had one more seizure on CSPI and 3 hospital admissions related to hypocalcemia with mild tetany in feet or hands. His creatinine concentrations have been stable, urinary calcium excretion normalised after first year of therapy, and nephrocalcinosis has not progressed. His development is normal, and he is growing normally. BMAD at the age of 3 yrs is within the age-adjusted pediatric reference interval (Fig. S4).

Patient 3

Presentation: Patient 3 was born to a mother with ADH1. Antenatal scans showed polyhydramnios. During pregnancy, her mother required temporary dialysis and developed seizures. She was delivered at the 31st gestational week by cesarean section for maternal reasons with a birth weight of 1.14 kg (3rd centile). Parents are of White European origin. She presented with respiratory depression at birth that required mechanical ventilation and was found to have severe hypocalcemia (serum calcium 0.76 mmol/L) and hypomagnesemia (0.52 mmol/L). Despite i.v. calcium boluses and infusion, she developed hypocalcemic seizures at the age of 5 days.

Before CSPI: She was treated with alfacalcidol, and calcium and magnesium supplementation (Fig. 1A), but still experienced multiple seizures and required frequent hospital admissions. She developed nephrocalcinosis and renal impairment with increased creatinine concentrations from 2 years of age and her GFR was 50-60 ml/1.73m^2/min in the years before start of CSPI. At the age of 18 years she commenced PTH(1-84) bolus therapy, but suffered hypocalcemic seizures whilst on once daily PTH(1-84) injections. She was switched to twice daily PTH(1-34) bolus injections in an attempt to improve this, and showed some reduction in the frequency of seizures and reduced hospitalizations. She had mild learning difficulties requiring special education. She completed normal growth.

After CSPI: She started CSPI at 22 years of age with an Ommipod patch pump and later changed to a Medtronic pump. She had one more seizure whilst on CSPI. Her adherance was suboptimal at times. Her nephrocalcinosis and GFR values have remained stable.

Patient 4

Presentation: Patient 4 was born at 39 weeks gestation after a normal pregnancy with a birth weight of 4.19 kg. Parents are of White European origin. He presented with recurrent seizures at 7 days of age, and was initially treated with phenobarbitone. He had marked hypocalcemia, hypomagnesemia, hyperphosphatemia, and undetectable circulating PTH concentrations (Fig. 1A). There was no family history of hypocalcemia.

Before CSPI: He had further recurrent left-sided focal seizures aged 5 weeks despite being on alfacalcidol, and calcium and magnesium supplementation (Fig. 1A), and received further treatment with phenobarbitone. He developed bilateral cataracts by 6 weeks of age, which were treated with lensectomy and glasses.

After CSPI: CSPI was started at age 7 weeks and his seizures completely stopped, he required 3 further hospital admissions due to intercurrent illness and infection of the infusion site. He has not developed nephrocalcinosis and his renal function is normal. His development, learning, and growth are normal. BMAD at age of 3 yrs is within the age-adjusted pediatric reference interval (Fig. S4).

Patient 5

Presentation: Patient 5 was born at 38 weeks gestation after induction of labour and emergency caesarean section due to polyhydramnios, with a birth weight of 4.15 kg. Parents are of Black African origin. She received 7 days of antibiotics for PROM and suspected sepsis but cultures remained negative. She had seizures at 9 days of age, and was initially treated with

phenobarbitone. She had marked hypocalcemia, hypomagnesemia, and hyperphosphatemia and low circulating PTH concentrations (Fig. 1A), and additionally hypoglycemia and hypokalemia. There was no family history of hypocalcemia.

Before CSPI: She was commenced on alfacalcidol, calcium, magnesium supplementation (Fig. 1A), which failed to resolve her seizures and she continued to be dependent on i.v. calcium treatment. She developed hypercalciuria and continued to have hypoglycemic episodes with an increased glucose requirement up to 11mg/kg/min. Further investigations were in line with hyperinsulinism of infancy (serum glucose 2.6mmol/L [3.0-7.8mmol/L], C-peptide 581pmol/L [370-1470], insulin 6.7 mU/L [2.6-24.9]). This was succesfully treated with diazoxide (maximum dose 5mg/kg/day) and hydrochlorothiazide. Analysis of genes related to congenital hyperinsulinism (*KCNJ11, ABCC8, AKT2, GLUD1, GCK, GPC3, HADH, HNF4A, INSR, KDM6A, KMT2D, SCL16A1, CACNA1D, PMM2, TRMT10A, HNF1A*) did not show any genetic variants. In addition, she has polyuria and polydipsia with hypokalemia (K^+ 2.8mmol/L [3.5-5.3mmol/L]), and on occasions mildy elevated plasma aldosterone and renin concentrations (aldosterone 706-1150 pmol/L [150-550], renin 1.4 - 10.9 nmol/L/h [<10.5]) consistent with Bartter syndrome type 5. She was continually in hospital for the first 6.5 months of life because of persistent hypocalcemia and her complex needs.

After CSPI: At 11 weeks of age she started CSPI, and i.v. calcium infusion could be weaned and stopped at age 15 weeks. Seizures completely stopped. She was discharged for the first time in her life at the age of 6.5 months. Since then, she has required 3 admissions for hypocalcemia mostly due to intercurrent illness and vaccination, occasionally accompanied by tetany. Diazoxide could be reduced to 2 mg/kg/day but attempts to stop have so far been unsuccesful. She has not developed nephrocalcinosis and her renal function is normal. She has achieved normal motor and language milestones. She is growing normally.

Patient 6

Presentation: Patient 6 was born at 35 weeks gestation to parents of White European origin with a birth weight of 3.46 kg. Antenatal scans showed polyhydramnios. She had hypocalcemic seizures at 6 days of age, in association with hypomagnesemia and low circulating PTH concentrations. There was no family history of hypocalcemia.

Before CSPI: She was treated with alfacalcidol, calcium supplementation and magnesium supplementation. Because of insufficient seizure control she was commenced on PTH(1-34) bolus injections at age 18 months and continued on magnesium supplementation which ameliorated but not stopped her seizures. She required increased bolus doses of PTH(1-34) to maintain target calcium concentrations. She had polyuria, polydipsia and hypercalciuria related to Bartter syndrome type 5, but did not have evidence for nephrocalcinosis. She had mild speech delay but attends mainstream school.

After CSPI: At age 6 years she was started on CSPI, with initial improvement and halved PTH dose (from 3.6 μg/kg/day to 1.4 μg/kg/day) and she has not had any further seizures. One year into CSPI she required multiple admissions due to episodes of hyper- and hypocalcemia and increased PTH doses up to 3.4 μg/kg/day. In view of suspected tachyphylaxis she was weaned of CSPI and re-started on alfacalcidol and calcium supplementation. This was supplemented with PTH(1-34) bolus injections at times of parental concern of hypocalcemia, which resulted in hypercalcemia (serum calcium 4.25 mmol/L) (Fig S3), and this necessitated emergency hospital admission. The tachyphylaxis could potentially have been due to neutralizing antibodies to PTH(1-34), which were not measured. However, the hypercalcemia resulting from over-treatment from PTH(1-34), suggests such neutralizing antibodies were unlikely to be present at high concentrations.

She re-commenced CSPI two months later due to severe episodes of hypocalcemia and deterioration of her renal function. Her calcium concentrations have been erratic and she has needed several admissions for hypo- and hypercalcemia in combination with hyper- and hypovolemia related to her Bartter syndrome type 5. At age 11 yrs she experienced left hip pain and was diagnosed with left slipped upper femoral epiphysis and underwent bilateral epiphyseal pinning. At the same age she was restarted on oral medication (cholecalciferol, alfacalcidol and clacium supplements) with the aim to wean CSPI due to difficult management, and worsening of renal insufficiency. Ultrasonography did not reveal evidence of nephrocalcinosis, but she has CKD stage 5 with a severely reduced GFR of 12 ml/min/1.73m^2, and is awaiting dialysis and renal transplantation. She has normal learning abilities, and growth is within the normal range.

Administration of PTH(1-34) by continuous subcutaneous infusion

PTH(1-34) (teriparatide) was administered using Medtronic Veo pumps in five patients (insertion areas with sufficient subcuaneous fat on the upper buttocks or upper thigh) and an Omnipod patch pump in one patient (insertion in the upper arm). Patients were admitted at the start of treatment. The pump reservoir was filled with PTH(1-34) (250 µg/mL) diluted 1 in 5 in normal saline (50 µg/ml). Medtronic Quick sets were used for tubing and cannula insertion for Medtronic pumps. PTH(1-34) infusion was commenced at a dose ranging from 0.1-0.6 µg/kg/day, and at an insulin pump rate of 0.2-2.1 units/hour. The lowest infusion rate of 0.025 units per hour on an insulin pump delivered 0.012 mcg per hour of PTH (with forsteo concentration of 50mcg/ml). A constant PTH infusion rate would be used for 24 hours. Alfacalcidol was stopped at the start of infusion and calcium supplementation was weaned and stopped during the first week. The reservoir and infusion sets were changed every 3 days, as described[4]. Patients and/or their parents were shown how to: dilute PTH(1-34); use the pump

including set changes and adjustments of pump rates; and also detect signs of hypocalcemia and hypercalcemia.

Monitoring

PTH(1-34) infusion rate was varied by a pediatric endocrinologist or general pediatrician (or adult endocrinologist for patient 3) with the aim being to achieve serum adjusted-calcium concentrations between 1.70-2.20 mmol/L and without clinical signs of hypocalcemia. Serum calcium was monitored in all patients at 5-14 day intervals initially, and less frequently thereafter in some of the patients. Occasionally, for further fine tuning, patients would receive one rate for 1-3 days and another rate, 0.012 mcg/hr higher or lower for the next day. If hypo- or hyper-calcemia developed then the pump rate would be altered usually by 1 step on the insulin pump (0.025 units, i.e. 0.012mcg PTH per hour), and for symptomatic hypocalcemia, additional calcium supplementation (1 mmol/kg) would be given once, and PTH rate increased temporarily by multiple steps until the serum calcium approached the lower limit of the target range. All patients were seen at 3 monthly intervals when they underwent biochemical assessment. The patients underwent annual renal ultrasound scans. DEXA scans were performed in the younger patients at the age of 3-4 years.

DNA sequence analysis

Sanger DNA sequencing of all coding exons and exon-intron boundaries of the *CASR* gene, utilising exon-specific primers (SigmaAldrich), the BigDye Terminator v3.1 Cycle Sequencing Kit (Life Technologies), and an automated detection system (ABI3730 Automated capillary sequencer; Applied Biosystems), was performed, as reported[5].

Cell Culture and transfection

HEK293T cells were cultured in high glucose DMEM (Invitrogen) supplemented with 10% fetal bovine serum. HEK293T cells stably expressing NFAT-RE (HEK293T-NFAT-RE) were generated by transfecting HEK293T cells with 100 ng/mL of the pGL4.30[luc2P/NFAT-RE/Hygro] construct (Promega) using Lipofectamine 2000 (Life Technologies), for 48h before selection using 100 μg/mL hygromycin. To generate a wild type (WT) CaSR construct the pEGFP-N1 vector (Promega) was modified, whereby the EGFP sequence was removed and replaced by a DYKDDDDK (FLAG) tag, resulting in a pFLAG-N1-*CASR* construct. Site directed mutagenesis, using the QuikChange Lightning Site-directed Mutagenesis kit (Agilent Technologies) and *CASR*-specific primers (Thermo Fisher Scientific), was used to generate the mutant *CASR* constructs. HEK293T or HEK293T-NFAT-RE cells were transiently transfected in clear 96-well plates (Sarstedt) with 300 ng/mL WT or mutant pFLAG-N1-*CASR* constructs, using Lipofectamine 2000 (Life Technologies). For SRE Luciferase reporter assays, HEK293T cells were transiently co-transfected with 300 ng/mL pGL4.33[luc2P/SRE/Hygro] construct (Promega). Successful transfection was confirmed by immunofluorescent staining for CaSR-FLAG. Briefly, transfected cells were seeded onto glass coverslips for 48h before fixing in ice cold methanol for 10 min. Cells were blocked in 1% BSA before CaSR-FLAG was detected using rabbit anti-DYKDDDDK antibody (Cell Signalling Technology, D6W5B) at a 1:2000 dilution, followed by anti-mouse Alexa Fluor 488 antibody (Thermo FisherScientific, A32766), at 1:500 dilution. Coverslips were mounted using ProLong Gold antifade mountant with DAPI (Thermo Fisher Scientific), and visualised on an Eclipse E400 fluorescence microscope, as previously described[5]. Protein expression was also confirmed using Western blot, as previously described[6]. CaSR-FLAG tag expression was detected using the HRP conjugated primary mouse anti-DDDDK antibody (AbCam, ab49763) at a 1:2000 dilution. Calnexin was used as a housekeeper, and detected using a rabbit anti-calnexin (Millipore,

Ab2301) primary antibody at 1:1000 dilution, and goat anti-rabbit secondary antibody (BioRad, 1706515) at 1:2000. Western blots were visualized using an Immuno-Star WesternC kit (BioRad) on a BioRad Chemidoc XRS+ system.

Luciferase reporter assays

Luciferase reporter assays were performed as described[7]. Briefly, 24 hours after transfection with WT or mutant CaSR constructs, cell growth media was replaced with starvation media (0.45mM $CaCl_2$, 0.01% FBS, 25mM HEPES) for 18 hours. Subsequently, cells were treated with 0.1-7.5mM $CaCl_2$ (≥99.0%, Sigma) for 4 hours at 37°C to induce SRE and NFAT-RE luciferase reporter expression, respectively. For studies with NPS 2143 (Sigma-Aldrich), cells were pre-treated with 100nM NPS 2143 or vehicle (DMSO) for 30 minutes at 37°C, and Luciferase reporter expression was induced in the presence of 100 nM NPS 2143 or vehicle. Following $CaCl_2$ stimulation, cells were lysed in Passive Lysis Buffer (Promega) for 30 minutes at 4°C and 5 minutes at room temperature. 20µl lysate of each well was transferred to 96-well white assay plates with opaque bottom (Corning). Luciferase intensity after the addition of 30µl firefly luciferase substrate (Promega) was measured using a PheraStar FS plate reader (BMG Labtech). Data analysis and statistical tests were performed in GraphPad Prism 8.4.0.

Statistical analysis

The statistical analysis plan did not include provision for correcting for multiplicity when conducting tests for secondary or other outcomes. Thus, the results of all analyses are reported as point estimates (arithmetic mean) with 95% confidence intervals. The widths of the confidence intervals have not been adjusted for multiplicity, therefore the intervals should not be used to infer definitive treatment effects for secondary outcomes. Biochemical data is

presented in Fig. 1 as box and whisker plots. *In vitro* data is presented as mean±SD of 4-6 biological replicates. Data analysis was performed in GraphPad Prism 8.

TABLES

Table S1. Urine calcium: creatinine ratio in six autosomal dominant hypocalcemia type 1 (ADH1) patients.

Patient	Urine Ca:Cr ratio on Ca+D (Pre-CSPI)	Urine Ca:Cr ratio on CSPI
1	3.7, 10.6[a] (n=2)	1.9 (1.7 to 2.2; n=88)
2	0.9 (0.7 to 1,2; n=9)	1.1 (0.9-1.3; n=30)
3	1.1, 1.1 (n=2)	1.0 (0.7 to 1.3; n=6)
4	2.1 (0.08 to 4.1; n=5)	1.2 (0.8 to 1.6; n=21)
5	1.5[a] (0.5 to 2.4; n=11)	0.3 (0.2 to 0.4; n=7)
6	4.4[a] (n=1)	1.1 (0.9 to 1.3; n=29)

Ca:Cr, calcium:creatinine. ratio; Ca+D, calcium and vitamin D analog therapy; CSPI, continuous subcutaneous PTH(1-34) infusion. Urine Ca:Cr ratios are represented as mean and 95% confidence interval (in parentheses). Individual urine Ca:Cr ratio values are shown for patients 1, 3, and 6 on Ca+D. The number of urine Ca:Cr ratio values per patient are shown (in parentheses). [a]Value >5 SD above the mean urine Ca:Cr ratio for the respective patient when on CSPI therapy.

FIGURES

Figure S1

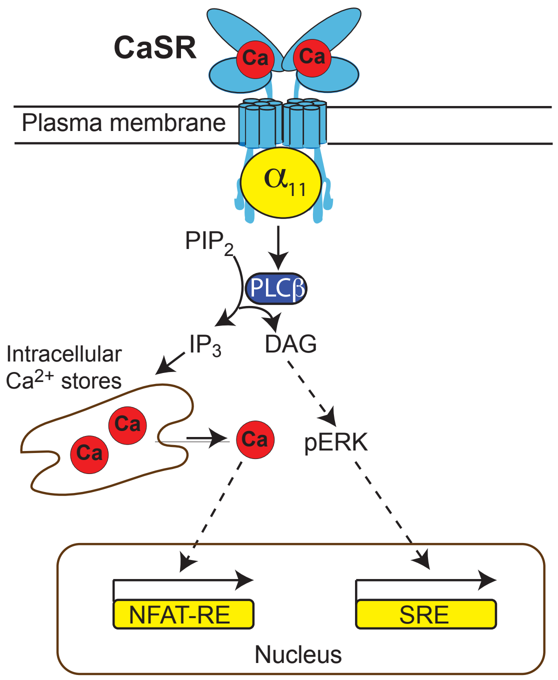

Figure S1. CaSR signaling pathway.

The binding of calcium (red filled-in circle, Ca) to the extracellular bilobed venus fly-trap domain of the CaSR (light blue) results in Gα_{11} (yellow)-dependent stimulation of phospholipase C-β (PLCβ) (dark blue) activity, which catalyses the formation of inositol 1,4,5-trisphosphate (IP_3) and diacylglycerol (DAG) from phosphatidylinositol 4,5-bisphosphate (PIP_2). An accumulation of IP_3 mediates calcium mobilisation into the cytosol from intracellular stores, whereas DAG activates the phospho-extracellular signal regulated kinase (pERK) arm of the mitogen activated protein kinase (MAPK) cascade. Gene transcription mediated by intracellular calcium and MAPK signaling can be measured using nuclear factor of activated T-cells response element (NFAT-RE) and serum response element (SRE) containing luciferase reporter constructs, respectively[5].

Figure S2

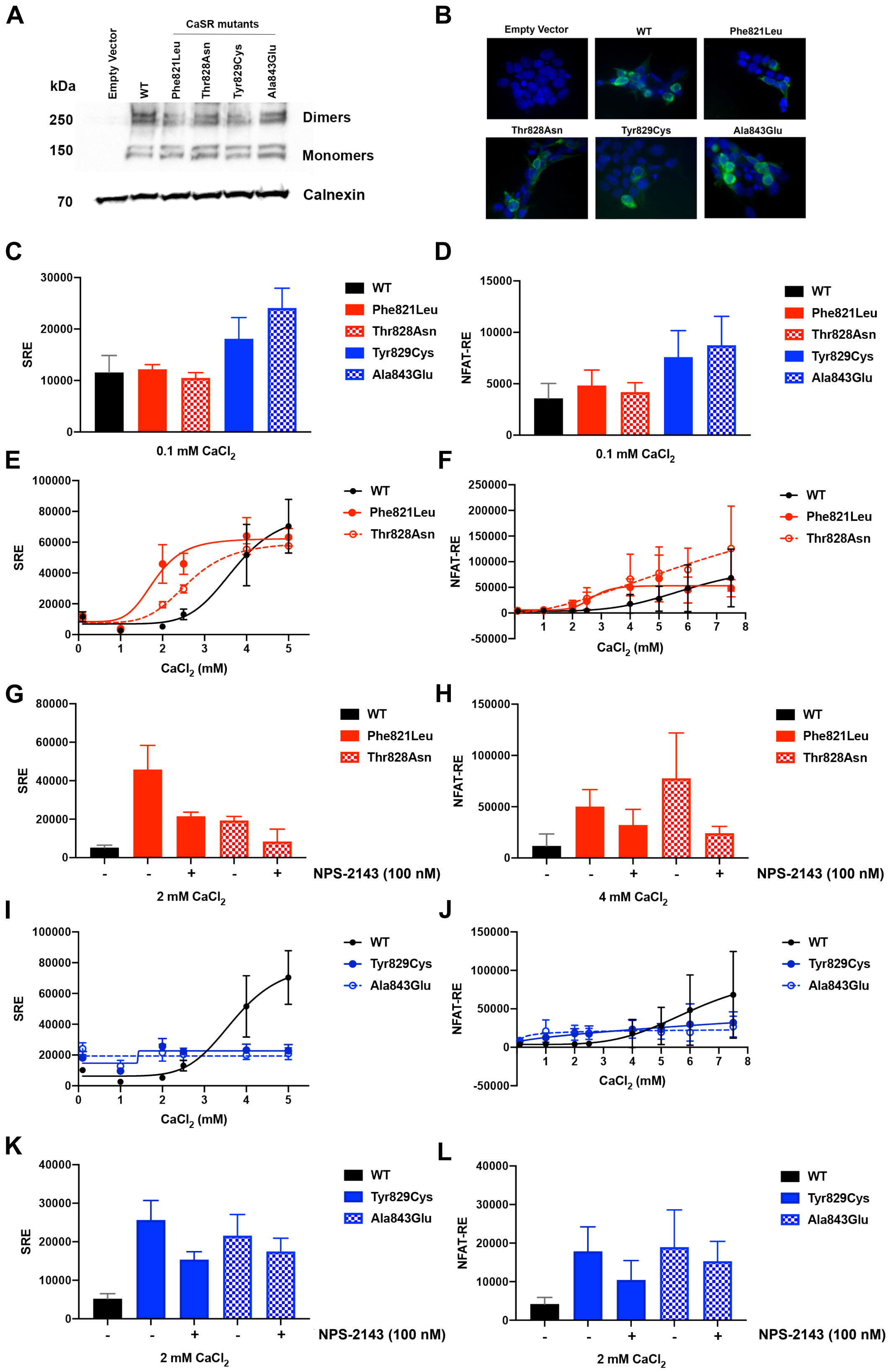
A
CaSR mutants
Empty Vector
WT
Phe821Leu
Thr828Asn
Tyr829Cys
Ala843Glu
kDa
250
150
70
Dimers
Monomers
Calnexin
B
Empty Vector
WT
Phe821Leu
Thr828Asn
Tyr829Cys
Ala843Glu
C
SRE
0.1 mM CaCl2
D
NFAT-RE
0.1 mM CaCl2
E
SRE
CaCl2 (mM)
F
NFAT-RE
CaCl2 (mM)
G
SRE
NPS-2143 (100 nM)
2 mM CaCl2
H
NFAT-RE
NPS-2143 (100 nM)
4 mM CaCl2
I
SRE
CaCl2 (mM)
J
NFAT-RE
CaCl2 (mM)
K
SRE
NPS-2143 (100 nM)
2 mM CaCl2
L
NFAT-RE
NPS-2143 (100 nM)
2 mM CaCl2

Figure S2. Functional characterization of ADH1-associated CaSR mutant proteins.

Panel A shows a representative Western blot indicating expression of the dimeric and monmeric wild type and mutant CaSR proteins in the HEK293T cells used to assess SRE and NFAT-RE responses; calnexin was used as a loading control. Transfection was also confirmed by fluorescence microscopy as shown in Panel B; CaSR expression is shown in green, with cell nuclei indicted by DAPI staining (blue). Panels C and D show the respective baseline SRE and NFAT-RE responses of the four CaSR mutants, measured at 0.1 mM extracellular calcium. The CaSR mutants p.Phe821Leu and p.Thr828Asn are non-constitutively activating (shown as solid and hatched red), while the p.Tyr829Cys and p.Ala843Glu mutants are constitutively activating (shown as solid and hatched blue), compared to wild-type (WT) (solid black). Panels E and F show the respective SRE and NFAT-RE responses of the constitutively activating CaSR mutants (p.Tyr829Cys and p.Ala843Glu) to increasing extracellular calcium concentrations. Panels G and H show the respective SRE and NFAT-RE responses of the constitutively activating CaSR mutants (p.Tyr829Cys and p.Ala843Glu) to NPS 2143 (100 nM) treatment (+) or drug vehicle (-). The p.Tyr829Cys and p.Ala843Glu constitutively activating CaSR mutants showed diminished or absent responses following administration of NPS 2143, thereby indicating that calcilytic treatment may potentially not be effective for treatment of ADH1 due to constitutively activating CaSR mutations. Panels I and J show the respective SRE and NFAT-RE responses of the non-constitutively activating CaSR mutants (p.Phe821Leu and p.Thr828Asn) to increasing extracellular calcium concentrations. Panels K and L show the respective SRE and NFAT-RE responses of the non-constitutively activating CaSR mutants (p.Phe821Leu and p.Thr828Asn) to NPS 2143 (100 nM) treatment (+) or drug vehicle (-). The p.Phe821Leu and p.Thr828Asn non-constitutively activating CaSR mutants responded to NPS 2143 treatment.

Figure S3.

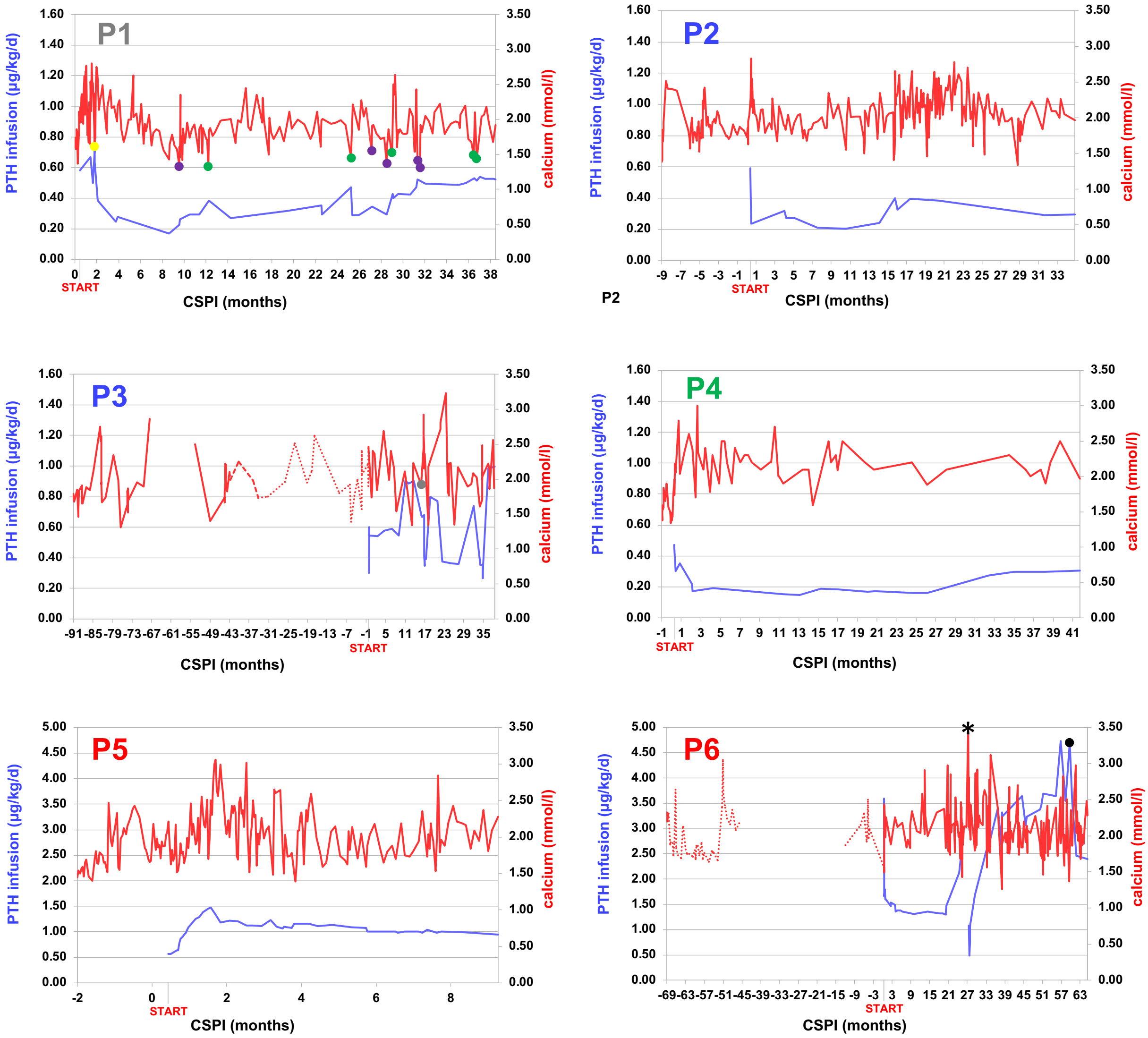

Figure S3. Effect of rhPTH(1-34) infusion on serum calcium concentrations in ADH1 patients Panels P1-P6 show serum adjusted-calcium concentrations (red line) in patients 1-6 before and during treatment with continuous subcutaneous rhPTH(1-34) infusion (CSPI), as well as the rhPTH(1-34) dose used in CSPI (blue line). For patients 1, 2, 4 and 5, data are shown from presentation and for patient 3 and 6, respectively, 7.5 and 5.5 years before start of CSPI. 'START' indicates start of CSPI. Before start of CSPI, treatment was with oral active vitamin D, calcium supplementations and/or i.v. calcium in patient 1, 2, 4 and 5, and PTH injections in patient 3 and 6. Dashed/dotted red line indicates serum calcium concentrations

during treatment with rhPTH injections [dashed indicates rhPTH(1-84), dotted line indicates rhPTH(1-34)]; gap (interruption) in the red line indicates absence of data]. For patient 1, hypocalcemic events are indicated: yellow dot indicates asymptomatic hypocalcemia without a cause, green dots hypocalcemia due to vaccination and/or intercurrent illness and purple dots due to pump cannula failure. For patient 3, the grey dot indicates when CSPI by OmniPod device was switched to Medtronic pump. For patient 6, interruption of the blue line indicates cessation of CSPI for suspected tachyphylaxis and the black dot indicates when the patient was re-started on conventional oral medication and CSPI weaned. *serum calcium concentration of 4.25mmol/L due to overtreatment with emergency rhPTH injection by parents. Note the difference in scale for PTH infusion dose for patient 1-4 vs 5-6.

Figure S4

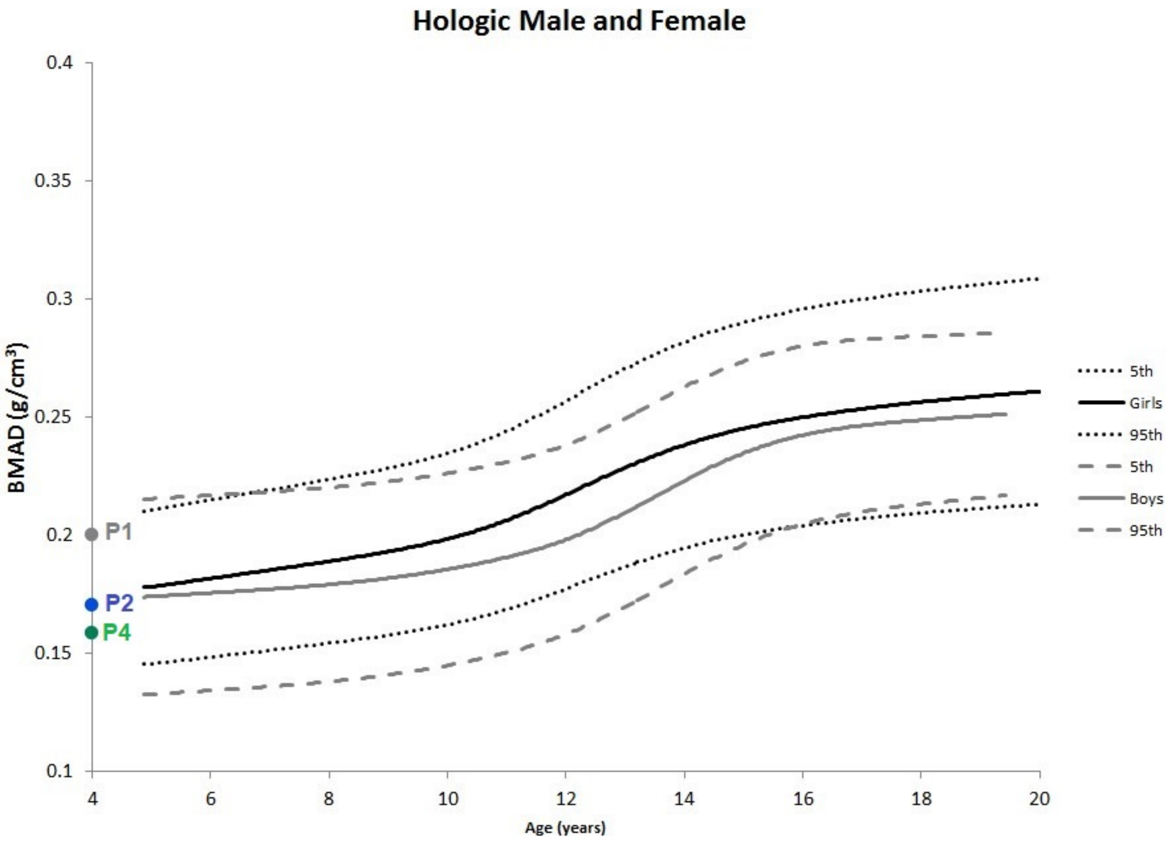

Figure S4 Bone mineral apparent density (BMAD) in three ADH1 children.

The solid and dotted lines represent the gender specific age-related BMAD reference intervals for a Hologic DXA scanner (mean, 5th and 95th centile) for 5-20 year olds[8]. BMAD reference intervals for children under the age of 5 years have not been established. Patients 1, 2 and 4 are represented in grey, blue and green. (Patient 1 BMAD 0.204g/cm^3 at age 3 years, patient 2 BMAD 0.167g/cm^3 at age 3.8 years, patient 4 BMAD 0.159 g/cm^3 at 3.6 years).

REFERENCES

1. Babinsky VN, Hannan FM, Ramracheya RD, et al. Mutant Mice With Calcium-Sensing Receptor Activation Have Hyperglycemia That Is Rectified by Calcilytic Therapy. Endocrinology 2017;158:2486-502.
2. Gray E, Muller D, Squires PE, et al. Activation of the extracellular calcium-sensing receptor initiates insulin secretion from human islets of Langerhans: involvement of protein kinases. J Endocrinol 2006;190:703-10.
3. Hough TA, Bogani D, Cheeseman MT, et al. Activating calcium-sensing receptor mutation in the mouse is associated with cataracts and ectopic calcification. Proc Natl Acad Sci U S A 2004;101:13566-71.
4. Linglart A, Rothenbuhler A, Gueorgieva I, Lucchini P, Silve C, Bougneres P. Long-term results of continuous subcutaneous recombinant PTH (1-34) infusion in children with refractory hypoparathyroidism. J Clin Endocrinol Metab 2011;96:3308-12.
5. Gorvin CM, Stokes VJ, Boon H, et al. Activating Mutations of the G-protein Subunit alpha 11 Interdomain Interface Cause Autosomal Dominant Hypocalcemia Type 2. J Clin Endocrinol Metab 2020;105.
6. Gorvin CM, Babinsky VN, Malinauskas T, et al. A calcium-sensing receptor mutation causing hypocalcemia disrupts a transmembrane salt bridge to activate β-arrestin biased signaling. Science Signaling 2018.
7. Howles SA, Wiberg A, Goldsworthy M, et al. Genetic variants of calcium and vitamin D metabolism in kidney stone disease. Nat Commun 2019;10:5175.
8. Crabtree NJ, Shaw NJ, Bishop NJ, et al. Amalgamated Reference Data for Size-Adjusted Bone Densitometry Measurements in 3598 Children and Young Adults-the ALPHABET Study. J Bone Miner Res 2017;32:172-80.
